# Primary Health Care Physicians’ Adherence and Attitude Towards Integrated Management of Childhood Illness Guidelines in Alexandria Governorate in Egypt

**DOI:** 10.5539/gjhs.v8n5p217

**Published:** 2015-10-20

**Authors:** Ahmed A. El-Ayady, Dorreya E. Meleis, Marwa M. Ahmed, Rania S. Ismaiel

**Affiliations:** 1 Department of Pediatrics, Cairo University, Cairo, Egypt; 2 Department of Community Medicine, Alexandria University, Alexandria, Egypt; 3 Department of Family Medicine, Cairo University, Cairo, Egypt

**Keywords:** under-five, Integrated Management of Childhood Illness, primary health care

## Abstract

**Background:**

Integrated Management of Childhood Illness (IMCI) is a cost-effective strategy that improves the quality of care provided to under-five children. Alexandria was the first governorate that applied the Integrated Management of Childhood Illness guidelines in Egypt. The aim of this study was to assess the degree of primary health care physicians’ adherence and attitude towards those guidelines after 17 years of application.

**Methods:**

This cross-sectional study was carried out on a representative sample from the primary health care facilities in Alexandria from which physicians using IMCI guidelines were included in the study. The sample units were chosen randomly from all districts of Alexandria. Observational checklists were developed to assess the degree of adherence of physicians based on the guidelines booklet.

**Results:**

The highest adherence score reported was that of writing disease classification (100%). As regards infants aged up to 2 months, the highest physicians’ adherence score reported was that of jaundice and possible bacterial infection assessment (100% and 95% respectively). And in spite of its importance, only 85.7% of physicians were complied with weight assessment and its plotting in the growth curve. For children aged from 2 months up to 5 years physicians were generally well complied with the guidelines especially for assessment of dangerous signs and possible bacterial infection.

**Conclusion:**

Despite being applied for years, IMCI guidelines still show certain areas of poor adherence, an issue that need further investigation in order to maximize physicians’ adherence and achieve the best of their performance.

## 1. Introduction

Practice guidelines have been developed by ministries of health, medical institutes and many local organizations in an attempt to reduce undesirable variation of health care and to improve its quality (Hyams, Shapiro, Brandenburg, & Brenan, 1996). Since the early 1980s symptom specific algorithms and training programs have been developed by the World Health Organization (WHO) and incorporated into the national health programs of many developing countries (Perkins et al., 1997).

The integrated management of childhood illness (IMCI) is one of these algorithms which were developed to address the most common causes of morbidity and mortality among children in developing countries (Bern, Zucker, & Perkins, 1997). The IMCI guidelines depend on case detection using few simple clinical signs and allow empirical treatment based on action oriented classification rather than the exact diagnosis (Gove, Tamburlilni, & Camphel, 1999; Gove, 1997).

IMCI implementation process consists of three phases: Introductory phase, early implementation phase and expansion phase. In Egypt IMCI was introduced in 1997 and Alexandria was the first governorate started applying IMCI guidelines in the primary health care units (Department of child and adolescent health development, 2010). IMCI has been implemented in 19 out of 26 governorates, in 102 out of 246 districts and in 1912 of a total of approximately 4500 health facilities in the country (WHO, 2010).

Family physicians are the main providers of care at the primary health care level. They are well oriented by all family members, their social, health and environmental backgrounds and have a good chance for proper communication and education for all family members. This facilitates achievement of the main goal of IMCI guidelines which is combination of both health care system and community practices in order to maximize the benefit of service provided at the primary level of care with reduction of both child morbidity and mortality (Harrison et al., 1993).

### 1.1 Objectives

In an attempt to assess primary health care physicians’ adherence to IMCI guidelines after 17 years of its application, this study has been carried out on a sample of primary health care facilities in Alexandria (Egypt).

## 2. Methods

### 2.1 Study Design

This is a Cross sectional descriptive study conducted in primary health care facilities in Alexandria from November 2011 to October 2012.

### 2.2 Research Setting

#### 2.2.1 Target population

Primary health care physicians who were using IMCI guidelines and agreed to participate in the research.

#### 2.2.2 Unit of Observation

Selected sample from all primary health care facilities in Alexandria districts.

#### 2.2.3 Sampling

A list of all the 73 primary health care facilities in Alexandria was obtained from the local health directorate. The number of units selected from each district was proportionate to the total number of units located there. Then finally the sample units were chosen randomly. The total number of included units was 37.

During the research visits, the researcher tried to include all primary health care physicians working in IMCI clinics in the study. One hundred physicians agreed to participate in the study and they were observed while examining 125 children (20 under age of 2 months and 105 aged from 2 months to 5 years).

### 2.3 Research Tools

To assess physicians’ adherence to IMCI guidelines: Two observation checklists were developed; one for each age group (zero up to two months and two months up to five years). Each checklist is composed of 4 sections:

- Assessment of sick child as recommended by IMCI protocol.
- Classification of disease based on the IMCI guidelines.
- Treatment given or identified including referral if needed.
- Mother counseling including feeding counseling according to the child’s age, when to return immediately, reason of referral if needed, giving referral note and giving health education about how to give oral drugs and teaching how to treat local infection if needed.

For each observation item: if it was not applicable score of zero was given, if it was done incorrectly score of (1) was given and if it was done correctly score of (2) was given. Then total score percentage for each part in the observation checklist was calculated to determine the mean adherence of physicians with each part in the IMCI guidelines according to the following equation (Abd El hameed, 2006):


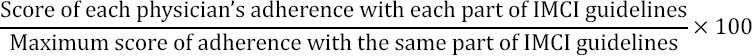


For assessment of physicians’ attitude towards IMCI guidelines, specially designed questionnaire was developed to assess physician’s attitude towards using IMCI guidelines. The content validation of the questionnaire was done by two experts. The questionnaire was tested on 30 physicians in order to check the clarity of the structured questionnaire and to estimate the time needed to complete the questionnaire. It includes 4 parts:

a. Specialty and the duration of using IMCI guidelines
b. IMCI advantages through five points scale ranging from strongly disagree to strongly agree. The advantages of IMCI guidelines were highlighted in fourteen points including: doctor – patient relationship, being organized system, ensuring proper child examination and non-missing serious illness, availability of drugs, organized system of working environment and referral. Then mean percentage of satisfaction was calculated and found to be (43.3%) which was accepted as a cutoff point to evaluate the attitude of physicians towards IMCI guidelines advantages.
c. IMCI disadvantages through five degrees scale ranging from strongly disagree to strongly agree. The disadvantages were highlighted in thirteen points including: IMCI is time consuming, IMCI needs special training, IMCI did not add anything as regards patient’s follow up, mother counseling and referral. Then mean percentage of physicians’ opinion towards IMCI disadvantages was calculated and found to be (18.25%) which was accepted as a cutoff point to evaluate the attitude of physicians towards IMCI guidelines disadvantages.
d. Physicians’ recommendations to improve IMCI implementation.

### 2.4 Statistical Analysis of Data

The collected data were organized, tabulated and statistically analyzed using statistical Package for Social Sciences (SPSS) version 16. Data were statistically described in terms of Mean, ± Standard Deviation (± SD) or Frequencies (Number of cases) and percentages when appropriate. Exact Fisher test was used instead when the expected frequency is less than 5. For comparing categorical data, Chi square (χ^2^) test was performed. P value less than 0.05 was considered statistically significant.

### 2.5 Ethical Consideration

Formal approval from the central directorate for research and health development was performed. Written approval was taken from the director of each primary health care facility in order to enable the researcher performing the field visit. And written consents were taken from each participant physician.

## 3. Results

From all the visited family health care facilities, only 100 physicians accepted to participate in the study. Most of them were family physicians (72%) while 20% of them were pediatricians. General practitioners represented only 8%. In Egypt, the graduated physician who doesn’t have any postgraduate studies is called general practitioner. All physicians have attended training workshop on IMCI guidelines.

### 3.1 Physicians’ Adherence With IMCI Guidelines

#### 3.1.1 Infants below 2 Months

The total number of observed clinical consultations in the IMCI clinics was 20. Eleven physicians referred to the chart booklet during clinical examination. All physicians asked about convulsions and inability to feed. Although, all physicians looked for signs of respiratory distress, 10% did not count the respiratory rate. All physicians were highly complied with the guidelines in checking for signs of infection, significant jaundice, diarrhea, assessing feeding problems and BCG immunization. In general, physicians had high adherence scores with the IMCI guidelines for this age group. Table 1 shows the difference in adherence scores among physicians depending on the type of health facility.

**Table 1:** Comparison between physicians’ means adherence scores of both accredited and non-accredited family health facilities

Adherence score	not accredited (n= 7)	Accredited (n= 13)	t p value
**Assessment of bacterial infection**	32.1± 0.8 (94.41%)	32.9 ± 1 (96.76%)	1.675* 0.00
**Assessment of diarrhea**	4.3 ± 3.9 (43%)	5 ± 0.4 (50%)	0.422* 0.03
**Assessment of feeding problems**	12.8 ± 1.9 (91.43%)	11.4 ± 0.9 (81.42%)	2.28* 0.04
**Counselling about oral drugs**	10 ± 1 (71.42%)	13 ± 0.5 (92.86%)	0.7* 0.01

* Significant t when p < 0.05.

**Disease classification:** All physicians wrote the disease classification according to the IMCI guidelines.

**Treatment and referral:** All sick infants received treatment and 3 of them needed further referral due to possible serious bacterial infection.

**Mother counseling:** The majority of physicians (90%) insisted to give the mother proper feeding counseling and all of them explained to the mothers when to return immediately. However, only 82.9% of physicians were complied with all elements of oral drug counseling.

#### 3.1.2 Children From 2 Months up to 5 Years

The total number of observed clinical consultations in the IMCI clinics was 105. None of the physicians used the chart booklet. All physicians were highly complied with the guidelines in checking for difficult breathing, diarrhea, malnutrition, immunization status and presence of any other problem. Only 94% of physicians asked about ear problems and 2.9% of physicians missed asking about convulsions. Weight for age was properly determined and plotted in only 69% of children. The mean adherence score of physicians were variable. The highest adherence score was that of assessment of dangerous signs 9.91± 0.5 (with maximum score 10), however the poorest adherence score was that of fever assessment as 56.2% of physicians were not complied with all elements of checking fever.

**Disease classification:** All physicians wrote the disease classification according to the IMCI guidelines.

**Treatment and referral:** All sick children received treatment and 10 of them needed further referral due to severe pneumonia.

**Mother counseling:** all mothers were offered feeding counseling and all physicians explained to them when to return. Only 41.8 % of physicians were complied with all elements of oral drug counseling. Table 2 summarizes the mean adherence scores of physicians while assessing children aged from 2 months up to 5 years.

**Table 2:** Summary of physicians’ adherence scores with IMCI guidelines (2 months to 5 years)

Assessment element	Mean adherence score	Total score	% adherence score
Checking dangerous signs	9.91 ± 0.52	10	99.1
Cough & respiratory distress	6.02 ± 3.94	10	60.2
Diarrhea	14 ± 0.0	14	100
Throat problems	9.8 ± 0.82	10	98
Ear problems	6.99 ± 3	8	87.37
Fever	9 ± 4.5	16	56.2
Malnutrition	6.4 ± 1.05	8	80
Immunization	3.82 ± 0.56	4	95.5
Check other problems	4 ± 0.0	4	100
Feeding counseling	16.65 ± 5.39	22	75.68
Drug counseling	11.79 ± 1.21	28	42.1

### 3.2 Physicians’ Attitude towards IMCI Guidelines

When the mean score for physicians’ satisfaction with IMCI guidelines was calculated it was 43.3. The score of 43 was considered to be the cutoff point below which physicians were considered to have negative attitude towards IMCI guidelines. On the other hand, score of 43 or more was considered as positive attitude towards IMCI guidelines.

**Figure 1:**
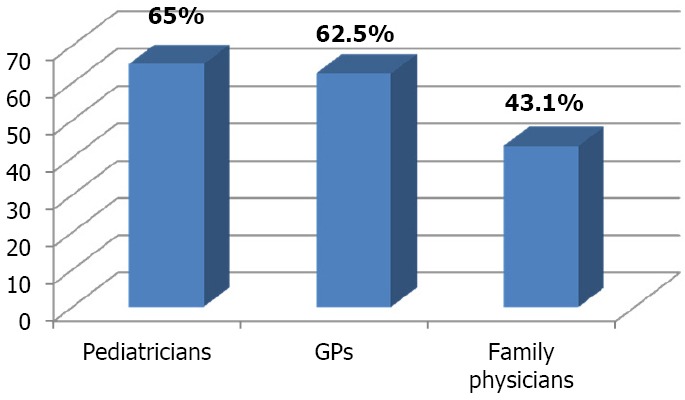
Distribution of physicians who had positive attitude towards IMCI guidelines according to their specialty

When physicians’ attitude was correlated with their specialty, it was found that pediatricians and general practitioners were satisfied with IMCI guidelines than family physicians and this was statistically significant (p value = 0.01). (Figure 1)

**Figure 2:**
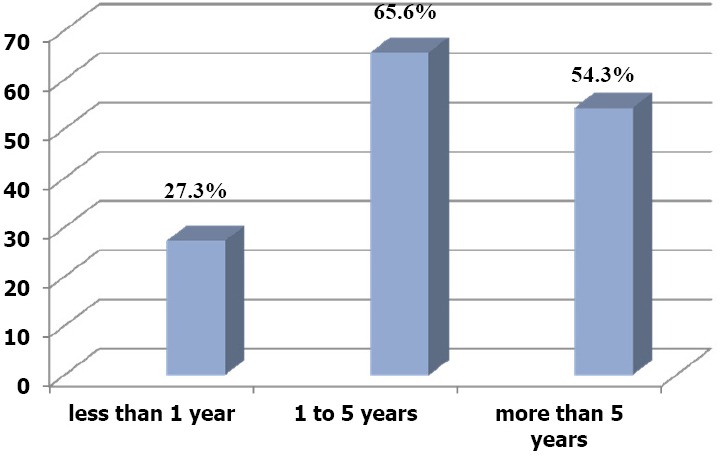
Distribution of physicians who had positive attitude towards IMCI guidelines according to the duration of practice

When physicians’ attitude was correlated to the duration of using IMCI guidelines, it was found that physicians who have been using the guidelines for less than one year showed statistically significant lower prevalence of positive attitude than others. (Figure 2)

**Figure 3:**
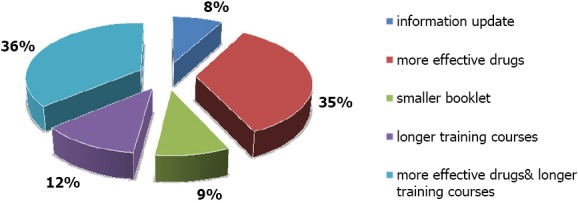
Physicians’ recommendations to improve health service in IMCI clinics

When participant physicians were asked about their recommendations to improve care in IMCI clinics they reported group of recommendations, which were summarized in Figure 3.

## 4. Discussion

As regards physicians’ adherence with IMCI guidelines for infants below 2 months: the majority of physicians (95%) were adherent to IMCI guidelines. This confirms the results of a report done by Department of Child and Adolescent Health and Development in 2000 to review the implementation process of IMCI which concluded that 98% of physicians followed the IMCI guidelines. The high adherence score may be due to the fact that physicians knew how critical this age group is and how important it is to assess carefully.

During assessment of physicians’ adherence with the guidelines for treatment and referral, 3 infants out of 20 needed further referral all of them due to possible serious bacterial infection this was also reported by study done in Gezira state (Sudan) which included that the main indication of referral was possible serious bacterial infection (Al Fadil et al., 2003).

The majority of physicians (90%) insisted to give the mother proper feeding counseling. All of them told mothers when to return immediately and the causes of referral when needed. The high adherence of physicians with mothers’ counseling is due to the high motivation of physicians to give educational messages to mothers especially for such critical age group. They also reported that counseling improves their own communication skills. This was also proven by a study done in Brazil which concluded that counseling messages covered by IMCI guidelines improved Physicians’ performances, maternal practices and some aspects of the diets and growth of children (Santos et al., 2001). However when adherence score for oral drug prescription was calculated it was estimated to be only 82.9%. This actually was due to missing of some points in the guideline especially: watching the mother measuring the dose herself and asking the mother to give first dose to the child. These 2 points considered not practical and time consuming to most of physicians.

In the present study 97.1 % of physicians checked three dangerous signs. Only 2.9% of them did not ask about convulsions. These results confirm the results of Health Facility Survey on Outpatient Child Care services (HFS) done in Egypt in 2002 by MOHP in collaboration with the Regional office for Eastern Mediterranean of WHO (EMRO) which reported that 94.9% of children were checked for three dangerous signs.

In a study done in Alexandria year 2002 to compare physicians’ performance in units applying IMCI and others not, the assessment of immunization status was done only in units applying IMCI guidelines (Abd El hameed, 2006). This complies with the results of the current study in which 91% of children were checked for their immunization status.

As regards the present study it was found that only 56 % of physicians follow the guidelines in checking all elements of fever assessment, which came in agree with the results of study done in Zambia from 1999 to 2000 to evaluate the effect of IMCI guidelines implementation on physicians’ performance (Edward & Phiri, 2002). An issue which needs further evaluation as poor assessment of fever in Zambia’s study was attributed to the initial implementation of the guidelines in such developing country however after 17 years of implementation in Alexandria it was expected for physicians to be adherent to such important element of children clinical assessment.

Being the provider, physicians’ attitude towards IMCI guidelines is definitely affecting their adherence and quality of provided care. In the present study the majority of physicians agreed that IMCI is an organized system that ensures no missing of serious illness mainly for young untrained physicians. However some physicians mentioned that IMCI is not new and its components such as referral, follow up and health education are well known and practiced in ordinary care without any need for a guideline. In the present study the highest positive attitude reported was among pediatricians as 65% of them had positive attitude towards IMCI guidelines compared to 43.1% of family physicians, they mentioned that they appreciate practicing through a guideline to prevent missing of serious conditions specially at the primary level of health care where physicians may not be trained enough or had limited experience, however in a study done in Morocco, it was found that pediatricians were less likely to accept IMCI guidelines than others. They rationalized that they had good clinical experience and did not need a guideline to miss serious diseases (Naimoli, Rowe, Lyaghfouri, Larbi & Lamrani, 2006).

In the current study when physicians were asked about their recommendations to improve health service provided in the IMCI clinics, one of the recommendations was the importance of having longer training workshops; they even reported that they want to have the same standard training program received by older physicians who joined IMCI program at its beginning. They stated that physicians who received the initial longer training program are more qualified, need less time during examination and do less effort checking the guidelines. Actually that was proven by a systematic review conducted by Rowe and his colleagues in 2011. The review reported that standard IMCI training course seemed to be more effective than shortened training one.

Other important recommendations of physicians in the current study were: the importance of having smaller easy booklet to carry, update of information and availability of health education materials such as printed cards for mothers to take home. These were also recommended by physicians in many studies before (Abd El hameed, 2006; Edward & Phiri, 2002).

Although being conducted on a representative sample from the primary health care facilities in Alexandria, this study suffers from some limitations. Physicians included in the study may show higher adherence scores than usual due to the fact of knowledge that they have been observed by the researcher.

## 5. Conclusion

The study aimed to assess the degree of physicians’ adherence to IMCI guidelines after been applied since 1999 in Alexandria (Egypt), it was found that although physicians still show certain areas of poor adherence, physicians who had been using it since its early beginning are well complied with the guidelines.
